# Editorial

**Published:** 1871-09

**Authors:** 


### Editorial.

### *Asiatic Cholera.*

Through the columns of some of our late European cotemporaries, we are apprised of the gradual advance of cholera, from Western Russia and Poland—where it appears to have become almost endemic during the last four or five years—through Prussia, from which its spread to England is only the next step, and that a brief one, in its progress, and thence its arrival upon our shores will be a matter of course.

The British Parliament has not considered it beneath its dignity to concert measures for the protection of the people against this dreaded pestilence, and on the 27th ult., the House of Lords commenced in earnest the discussion of these measures. In view of the location of our city as an important depot in the great immigrant route from east to west, and the further fact that it has suffered so often and so fearfully in former years from this scourge, and, moreover, that during the past year nearly every emigrant ship from Germany has reached our shores laden with the virus of small pox, and that nearly every case of that disease which has occurred in the city has been directly traceable to that source, would it not be well for us to wake up from our apathy, and take measures to protect ourselves, before the time when our judgment shall abandon us under the influence of panic?

Meanwhile we would like to ask, what is the Board of Health doing? and fortunately the question is easily answered. They are trying to solve a problem attempted by the Israelites in Egypt three thousand years ago, *i. e.*, making bricks without straw. If, now, the same hope existed, as then, of the advent of a second Moses, under whose leadership they should "spoil the Egyptians," there would be consolation in the hope, but we fear "cum duplicantur lateres, Moses" non "benit."

The Board of Health has borne much undeserved odium, heaped upon it by the secular press, and others, and it would be as well, before adding to its load, to inquire into the causes which underlie its alleged inefficiency.

We have neither the time nor inclination to go into the details of these causes. Those of our readers who are so disposed, can readily do so by consulting the proceedings of the Common

Council. But this we will say, without hesitation and from positive knowledge, that should this city be visited by cholera, or any other pestilence, from a lack of proper preventive and protective measures, the Common Council, and it alone, will be responsible.

Amongst all the charges that have been urged against the Board of Health, political prostitution has never been one, and we honestly believe its skirts to be free from the stain. The Board is not a political organization, and, we believe, cannot be used as such; hence the Common Council fails to perceive its utility, and obstinately and stupidly continues to obstruct its operations by refusing the necessary funds for their conduct. We say obstinately and stupidly, because their refusal is in gross violation, not only of the spirit but also of the letter of the law, under which the Board of Health exists, and this, too, when the expenditures of the Board during the past year have been less than ever before—less, even, than under the old political regime, when the city had one-third of its present population.

In times of public panic from pestilence, citizens look to their physicians for protection, and we call upon the profession at large, and of the city in particular, to sustain the Board of Health in their honest efforts to protect their city against this hydra-headed monster of ignorance and obstinacy, the Common Council. W. H.

***Turkish Baths.***

Within the last few days, a handbill has been distributed to private residences in the city, advertising the advantages of a bathing establishment. in which handbill, under the heading "Medical Testimony," appears the following "from the *Chicago Medical Journal*":

"The simple action of the Turkish Bath is to *equalize the Circulation*, *remove Congestion*, *distribute Nutrition*, restore and keep active all the secretions, whether of the skin, the kidneys, or the liver, and thus eliminate poison from the system.

"And any experienced physician will be able to see how many diseases may be benefited or entirely controlled by such an instrumentality.

"Over and above all, the Turkish Bath *purifies the blood* more directly and thoroughly than any other means the science of medicine has ever before suggested. Perspiration eliminates the water from the blood loaded with the waste and poisonous matter from the system; and by drinking cold water the blood is replenished with wholesome material in exchange. Indeed, this is the most important purpose which perspiration serves. Man perspires because perspiration being the watery portion of the blood, it carries off with it all of the extraneous and poisonous matter; thus removing all constitutional taints, whether of *Scrofula, Cancer, Syphilis, Tuberculosis, or any blood disease.*"

Now, to say that the endorsement above quoted never appeared in this Journal, would be to give "the lie direct," and we have too much respect for Touchstone's good counsel "to go further than the lie circumstantial." Therefore we say that such a mass of etymological nonsense, physiological ignorance and vulgar quackery could have disgraced its editorial pages only at one period in its history, when, at a date which, as Cervantes says, "no quiero acordarme," there was attached to the editorial staff of this Journal, in a capacity which would probably be designated by his own appropriate patronymic, an individual who seemed, by general consent of the profession, to be deemed qualified for retirement, without rank or pay, and who subsequently, we believe, rose gradually to some sort of laundry business.

We do not propose, just now, to discuss Turkish baths in general, further than to express our regret that, notwithstanding its alleged efficacy for "the removal of all constitutional taints," it should have so evidently failed to remove the taint of mendacity.

As to this particular notice, so far as we can understand it, it is a gross fraud upon the JOURNAL and the public.

We say as far as we can understand it, for we confess ignorance as to what is meant by "distribute nutrition," "restore and keep active secretions," etc. These alleged advantages may or may not be true; they are beyond our comprehension.

If "over and above all, the Turkish bath purifies the blood," it is a great pity that its usefulness might not be extended to the purification of the conscience of the manufacturer of the impertinent handbill above quoted. If Turkish baths will accomplish this, we will give them a hearty *editorial* endorsement. W. H.

*Editors of the Journal:*

Will you be so kind as to insert the following notice in the JOURNAL?

I believe the society will really establish a Medical Library worthy of Chicago.

The efforts of the committee for the present will be directed to collecting files of journals, proceedings of societies, etc.

The society has secured room in the fire-proof building of the Young Men's Christian Association, the librarian of which will perform all the labors connected with the care of books, etc.

If the JOURNAL will donate to the Library any old copies which may exist in duplicate, it will aid our efforts.

Most truly, E. L. HOLMES,
Chairman of the Committee.

NOTICE.

The members of the Chicago Medical Society are making earnest efforts to found and support a public Medical Library.

They have already secured liberal donations, and most ample and permanent accommodations in the fire-proof library building of the Young Men's Christian Association.

Will members of the profession throughout the Northwest, aid the society in obtaining a complete file of the various medical periodicals of our country, by sending *any* copies of such journals to the library? Old catalogues and announcements of medical colleges, hospital reports, etc., are desired.

Small packages may be sent by mail; large ones may be forwarded by express, "Care of Librarian of the Young Men's Christian Association," who will pay all express charges.

The library is open for all visitors from 9 A M. till 10 P. M.

LIBRARY COMMITTEE OF THE CHICAGO MED. SOC.

In directing the attention of our readers to the above communication, we, at the same time, cordially urge their co-operation in aiding the accomplishment of this laudable project, than which none may prove more advantageous to the profession at large, and to its younger members especially.

The want of a good library of reference is frequently an unsurmountable obstacle to the progress of many a would-be earnest worker. Few young men in our profession are blessed with the wherewithal to acquire this indispensable aid to study, and when the means for its acquisition are attained, too often professional ambition is lost, and he who might have become a brilliant ornament to his profession, has degenerated into a mere routinist, or possibly even a real estate operator.

Few are aware of the sort of material out of which a good working library can be constructed. Vast endowments, elegant book cases, and costly volumes are not essential, at the beginning at least. Every physician has in his own collection odd numbers of journals, duplicates, pamphlets, catalogues, etc., which are, to him, mere rubbish and worse than useless.

If each will contribute his stock of such matter, a judicious librarian will astonish the uninitiated by the amount of literary treasure he will extract from this dross.

We ask, on behalf of the committee, this much from all, as it is within the ability of all, but, at the same time, we trust that there are many who will aid the committee by substantial donations of books and money.

The committee is only known to us through its chairman, whose name alone, however, is a sufficient guarantee of good faith of the project. W. H.

***To Correspondents.***

Dr. Jas. D. Whitely, Oakford, Ill., writes to the JOURNAL under date August 2nd, enclosing specimen of diseased liver and omentum removed from a patient, and requesting our opinion as to the character of the morbid growth.

Upon microscopic examination, the organs appear to have sustained fatty transformation. The normal tissues, in the case of the liver, have been converted into fat, and in the omentum the areolar tissue to have become distended with fat; rather an hypertrophy of adipose tissue. W. H.

